# Eight practices for data management to enable team data science

**DOI:** 10.1017/cts.2020.501

**Published:** 2020-06-23

**Authors:** Andrew McDavid, Anthony M. Corbett, Jennifer L. Dutra, Andrew G. Straw, David J. Topham, Gloria S. Pryhuber, Mary T. Caserta, Steven R. Gill, Kristin M. Scheible, Jeanne Holden-Wiltse

**Affiliations:** 1 Department of Biostatistics and Computational Biology, University of Rochester, Rochester, NY, USA; 2 Clinical and Translational Science Institute, University of Rochester, Rochester, NY, USA; 3 Department of Microbiology and Immunology, University of Rochester, Rochester, NY, USA; 4 Department of Pediatrics, University of Rochester, Rochester, NY, USA

**Keywords:** Data analysis, pediatric, systems biology, bioinformatics, databases, data science, data management, research informatics

## Abstract

**Introduction:**

In clinical and translational research, data science is often and fortuitously integrated with data collection. This contrasts to the typical position of data scientists in other settings, where they are isolated from data collectors. Because of this, effective use of data science techniques to resolve translational questions requires innovation in the organization and management of these data.

**Methods:**

We propose an operational framework that respects this important difference in how research teams are organized. To maximize the accuracy and speed of the clinical and translational data science enterprise under this framework, we define a set of eight best practices for data management.

**Results:**

In our own work at the University of Rochester, we have strived to utilize these practices in a customized version of the open source LabKey platform for integrated data management and collaboration. We have applied this platform to cohorts that longitudinally track multidomain data from over 3000 subjects.

**Conclusions:**

We argue that this has made analytical datasets more readily available and lowered the bar to interdisciplinary collaboration, enabling a team-based data science that is unique to the clinical and translational setting.

## Introduction

Data science has appeared only recently as a distinct discipline [1]. Although it is often understood to mean the art and science of curating and analyzing data, another reading of the phrase is the use of pre-existing data to conduct science, as opposed to conducting experiments, or deriving theory. This latter reading directly implicates translational and clinical science as core domains of data science, as empirical disciplines that must heavily use observational data. Another hallmark of translational and clinical science is the diversity of expertise required, which has been deemed “team science.” Here we focus on how both the appropriate use of databases and human resources to administer them facilitates what we are calling Team Data Science.

Prospective, observational studies on human cohorts shed light on mechanisms of disease by generating novel hypotheses in ways that animal models cannot. For instance, despite improvements in survival for preterm, and low-birthweight babies, they remain at risk for multiple complications. Over 50% of them will be discharged with ongoing postnatal growth failure [2], whereas infants born before 27 weeks gestational age have a 1.5-fold increased risk of hospitalization for asthma later in life [3] compared with those born closer to term. Recent studies suggest that the infant gut and nasal microbiomes, potentially interacting with the immune system, directly impact growth and respiratory health [4]. However, these systems produce complex and high-dimensional data, such as that from sequencing or flow cytometry. Inevitably, predictive, and perhaps only phenomenological models will need to be developed before the mechanisms that generate the associations between microbiotic state, growth, and respiratory health are fully understood.

Data science and the “algorithmic modeling culture” have excelled at finding accurate predictive models, as well as providing techniques to organize complex data sets [1]. They have been contrasted to the “data modeling culture” of statistics that seeks or assumes knowledge of a data-generating process [5]. To effectively use data science techniques to resolve these translational questions requires innovation in the organization and management of these data. For over a decade the informatics team of the University of Rochester Clinical and Translational Science Institute’s Research Data Integration and Analytics group has been developing comprehensive data management workflows for laboratory assays, specimen inventories, and study-specific data using the open-source LabKey platform [6–10]. Early funding for this effort came from several NIAID grants that recognized the need to develop a system to manage and integrate high-throughput genomics and related data from human subjects.

As clinical and translational researchers our primary goal is to derive knowledge that can make useful predictions in other settings and secondarily, do this efficiently. Over time, the LabKey platform, our innovative customization of it, and our processes have matured. The data management system has become more than solely a database and data archive: it functions additionally as a central study and lab portal, aiming to improve data collection and reporting, analysis transparency, and rigor. These serve to increase the accuracy and velocity of clinical and translational science.

## Methods

### A Schematic for Team Data Science

In their book “R for Data Science” Wickham and Grolemund [11] introduce a schematic representing the workflow of a *data scientist*, with a data scientist-centric view. The data scientist, which we will refer interchangably as the *data analyst*, is someone skilled in collection, processing, visualization, modeling, and interpretation of large quantities of heterogeneous data, including both data at hand and data they acquire through ingenuity. They can be trained in different quantitative disciplines including statistics, epidemiology, computer science, and bioinformatics [1]. The analyst workflow begins with *importing* and “*tidying*” the data. Then the analyst iterates between *transforming*, *visualizing*, and *modeling* the data, until they *communicate* final results to stakeholders.

**Fig. 1.**
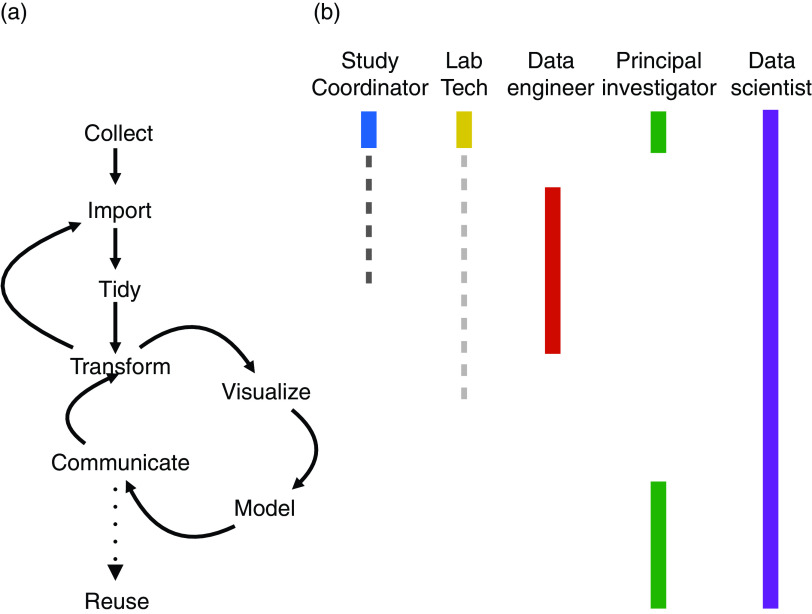
A data science workflow in clinical and translational teams. The lifecycle of a Team Data Science project begins with data collection and proceeds in a nonlinear and iterative fashion until conclusions are communicated and data and models are available for reuse (1a). Study personnel will interact in varying degrees with different aspects of the data science lifecycle (1b), while a data scientist visits all phases. Bolded interactions highlight a primary use of a role, while dashed lines indicate ancillary uses.

In the translational and clinical setting, data science follows a similar schematic but with some important modifications (Fig. 1a). First, the process will be rooted with the *creation and collection* of the data by a principal investigator launching a study, hiring study coordinators, and finally recruiting study participants. Because grant monies have limited time horizons, for observational studies, the analysis of the data often must begin while data collection is ongoing. Therefore the former two roles must be actively involved throughout data analysis.

We also add to Wickham and Grolemund’s diagram several other connections between steps they described. Since the data evolves as more study participants are recruited and data quality is evaluated, the import–tidy–transform steps now form an iterative cycle. This has implications for staffing and the technical frameworks used by analysts and *data engineers*. We define this latter role as encompassing both developing databases and pipelines as well as continual oversight of the quality of data sets. Data engineers must have clear lines of communication with both analysts and principal investigators to include useful transformations upstream for reuse. And since the data is evolving, it needs to be automatically ingestible by the platform and versioned and traceable by the analysts. This sort of cyclic evolution has been sometimes called the *slowly changing dimensions* (SCDs) of a data warehouse [12], with a particular set of methodologies to accommodate current and historical reporting and analysis.

We also include communication as part of the “transform–visualize–model” loop. In any domain of data science, business or scientific expertise must inform visualization and modeling. In the Team Data Science regime, it is expected that as scientific investigators parse results of a model, and their understanding of the science evolves, modifications to the transformation, modeling, and visualization will be made. After discussion with the study coordinator, the data scientist may end up dramatically altering a model, such as to make it better reflect causes of missing data or eliminate variables that are suspected to suffer from high levels of measurement error.

Finally, the ultimate goal of Team Data Science is to advance scientific knowledge. Therefore the data and interim research products must be *reusable* both within and across institutions. Wilkerson *et al*. [13] described this by stating that data must be FAIR: Findable, Accessible, Interoperable, and Reusable. Throughout the collecting and importing processes, modifications must be made to accommodate the requirements of FAIR data. However, interoperability and reuse seem to be best understood along a continuum. Data standards for interoperability are domain specific and frequently evolving, and the exact implementation is beyond the scope of any single article.

Wickham and Grolemund’s schematic is addressed to data analysts, who may be orphaned from the provenance of the data. However, as translational and clinical researchers, we rely on the effort of many different team members to accomplish our scientific goals (Fig. 1b). Besides the principal investigator, study coordinators, and data engineers described above, other personnel include lab technicians, clinicians, statisticians, and bioinformaticians, each of whom play lead roles (shown in bold in Fig. 1b) in various steps in the data analysis workflow. Moreover, these players also often end up using subsets of the Wickham and Grolemund diagram (shown in dashed lines) to accomplish their own discrete objectives. For instance, although a lab technician is primarily involved in collecting data, they may also need to import, tidy, and visualize their data in order to calibrate lab equipment or understand if positive and negative controls have behaved properly. These import–tidy–visualize steps could occur on an ad hoc basis or better yet, be explicitly included in the import–tidy workflow that a data engineer uses.

### Eight Practices to Implement Team Data Science

Policies, procedures and technical decisions about how data are stored and represented are required to concretely execute a theoretical framework such as that described in Fig. 1. In Table 1, we propose eight policies and procedures that help operationalize Team Data Science projects. We believe adopting these practices increases the speed and accuracy of data science on translational and clinical studies and describe how we have applied them to our own projects in the Results.

**Table 1.** Eight practices to implement team data science

Practice	Example
1. Active collaboration	Data engineers and analysts meet regularly with data collectors and domain experts
2. Consistent schema, field names, and identifiers	Data engineers introduce appropriate names and formats for study variables
3. Continuous quality control	Data evaluated for internal and external consistency and quality continually and automatically
4. Versioning, access control, and auditing	Users have differential privileges to read and change data. Changes are tracked and can be replayed.
5. User-driven data exploration	Charting tools are provided for quick and independent exploration of data
6. Import-derived variables	Variables derived by team members are published in central database
7. Defined data export format and programming interfaces	Data are available easily and scriptably in open formats
8. Online documentation	Documentation for data and pipelines is placed near to the means to access them

These practices recognize the presence of benefits and guard against some pitfalls that are central and perhaps unique to data science. Data science places value on rapid prototyping to test hypotheses and models. Secondly, it is expected that the data, which are costly and precious, should be used maximally in models and visualizations. This can be surprisingly challenging in practice, when disparate experiments and domains are being integrated. Having defined schema, changing data capture processes, continuous data quality control and defined data export reduce these problems.

Reuse of data and analysis also implies that findings must be internally reproducible: recomputable given the dataset and data analysis pipeline. However, findings should also be externally replicable such that an “independent experiment targeting the same scientific question will produce a consistent result” [14]. The iterative processes detailed in Fig. 1a tend to work in favor of reproducibility. It is only possible to run them efficiently in automated format, hence reproducible pipelines. However, without care, the iteration of the “model–visualize–transform” loop will damage external replicability. Iterative modeling and communicating introduce many “researcher degrees of freedom” [15], whereas the high-dimensional characteristics of the data mean that in the absence precautions, overfitting the data is inevitable. Fortunately, a potent remedy exists for this in data science by utilizing procedures that hold out portions of the data from the “transform–model–communicate” loop, via cross-validation and related techniques, in order to provide unbiased validation of accuracy and effect size.

## Results

We implemented the practices in Table 1 to collect and manage data for two large observational, prospective studies following 397 infants from birth to assess prematurity and respiratory outcomes: Prematurity and Respiratory Outcomes Program (PROP) [16] and the Prematurity, Respiratory outcomes, Immune System, and Microbiome Study (PRISM) [17]. The PROP Study was a multi-center study, with data managed locally for the 146 infants enrolled at the University of Rochester and followed for 1 year of life with frequent sampling during hospitalization and after discharge. The PRISM enrolled 267 infants who were followed with daily clinical respiratory, weekly sampling during hospitalization, monthly after discharge until 12 months corrected gestational age, and during respiratory illnesses in the first 2 years of life. The Research Subjects Review Board approved the studies and all parents provided informed consent (RSRB00037933 and RSRB00045470).

Over 1900 clinical and assay data fields were managed on these subjects, including up to 190 repeated measures per subject. Our data ranged from respiratory and nutritional data collected daily during the NICU hospitalization, to over 76,000 biospecimen vials which were managed in specimen inventory software. Multiple high-throughput and high-dimensional assays also generated data. These assays included flow cytometry, rt-PCR, Luminex, sequencing of mRNA, the 16S rRNA microbiome, virome, and exome, and respiratory inductive plethysmography.

**Fig. 2.**
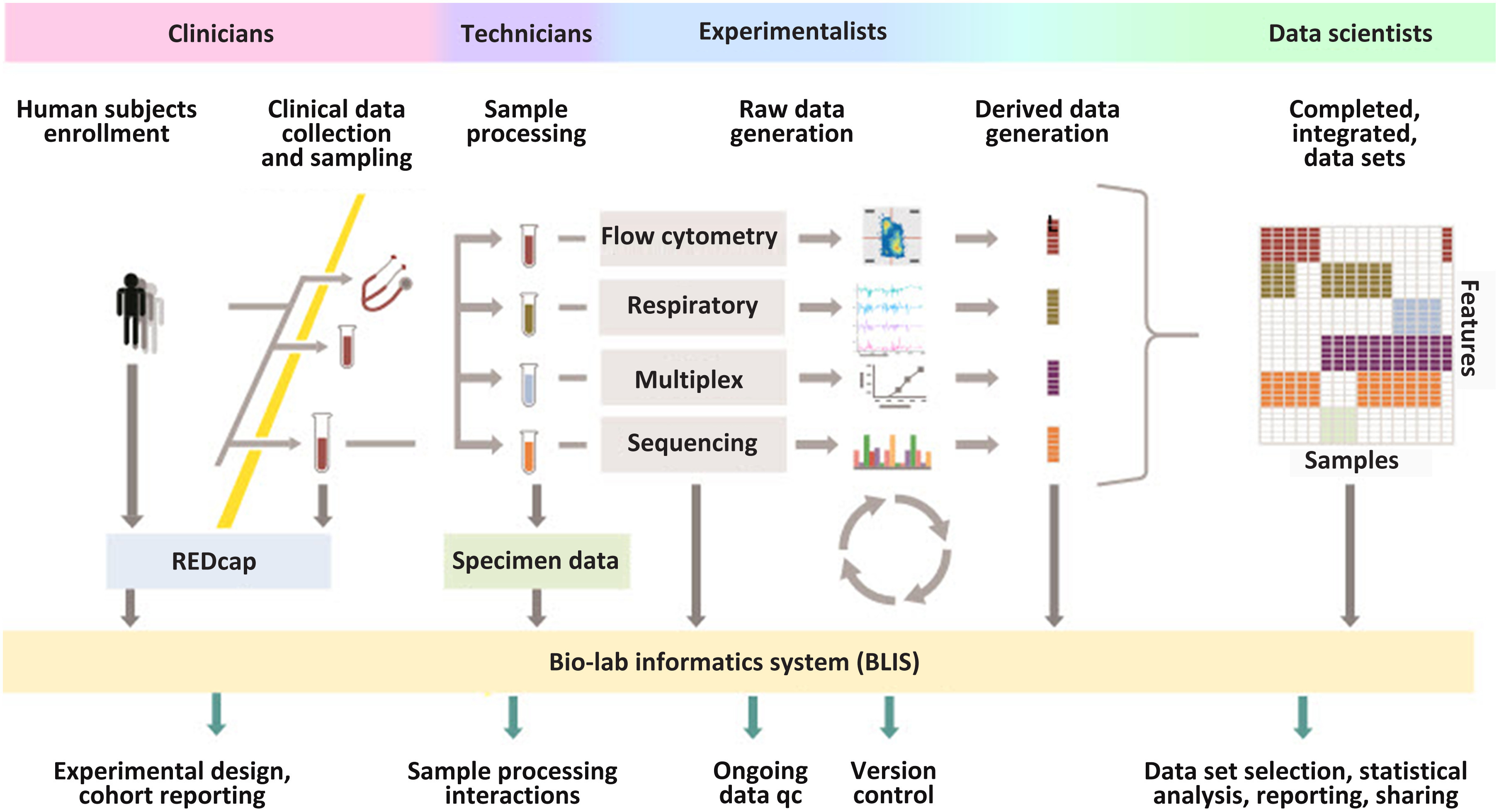
A high-level overview of how study personnel interact with the BLIS data management platform. Clinicians, technicians, and experimentalists generate data for different aspects of the study. Data engineers implement the centralized study portal using the BLIS data management platform, with responsibility to connect all elements of the workflow and interact continuously with all study team members.

We set up a central study portal which provided nightly automated data ingestion from the disparate data sources using our Bio-Lab Informatics System (BLIS) (Fig. 2). BLIS is a customized instance of the open-source LabKey [6–10], an application developed to integrate, analyze, and share biomedical research data, including flow cytometry, proteomics, Luminex, ELISpot, ELISA, Nab, rt-PCR, other plate-based assay data, as well as specimen inventory and clinical subject data (Fig. 2). The BLIS platform provides a secure relational database and web-based tools for interactive querying, visualizing, and sharing data across a range of data sources. We implemented pipeline modules to collect and parse assay data and scripts to validate and process experimental data and generate custom reporting. REDCap [18] was used to collect clinical and environmental exposure data by the clinic staff, and the sample-processing technicians entered specimen information into a third-party inventory application. Experimentalists and lab technicians uploaded raw instrument data output files and derived assay results into BLIS-developed assay modules.

Next, we describe some of the considerations and anecdotes we discovered in attempting to implement best practices for Team Data Science (Table 1). We believe addressing these practices was instrumental in the publication of 12 papers from these studies [4,17,19–27]. It also enabled us to easily adopt subsets of the data for use in class projects in courses we have taught.

First, data engineers and scientists should have active collaboration (practice no. 1) with data generators at all stages of a translational data science project. This ensures that the database is designed to appropriately capture the breadth of raw, intermediate, and final data products and their metadata to sufficiently enable downstream discovery, modeling, and reuse.

We find this ultimately is a question of resources, priorities, and study infrastructure. We include at least 50% full-time equivalent funding for data engineering for the study duration to provide ample time to attend study team meetings and develop study- and lab-specific data collection modules and reporting. This collaboration also extends in the other direction, with domain experts actively participating in data analysis plans, derivation of additional variables, and interpreting modeling outputs.

In BLIS, we further facilitate active collaboration using the wiki-style web pages, file sharing, and task tracking functions. In particular, the file sharing and wikis have been used to communicate study protocols and laboratory standard operating procedures, track decisions made around database design and changes, as well as plan and execute various data analyses. For the PROP and PRISM studies, we were able to harmonize the activities and communication of the 40 clinical, lab, and analyst team members.

Second, the database schemas, field names, and identifiers should be used consistently (practice no. 2) across database tables and table views. The schemas describe which fields belong to the tables, their data types or bounds, key types, and uniqueness constraints. The field names are the user-visible and internal names for various variables (columns) in the database. Put simply, these principles insure that tables in the database contain “tidy data”: each variable is a column, each observation is a row, and each type of observational unit is a table [28]. They are a prerequisite to any modeling or visualization that joins tables, therefore experiments or data modalities. Besides the analytic benefits, semantic consistency and uniqueness of identifiers provide a shared language between data collectors, engineers, and analysts. Data collectors will have their own preferred formats for collecting data, but consistency of identifiers is still possible and highly desirable.

In the PROP and PRISM studies, our primary identifiers were *participant ID*, *visit number*, and *date*. For each participant, a defined sequence of study events (clinical visits, sample collection, etc.) was to occur on various dates after their birth. *Visit number* indexed this sequence, tracking compliance with the protocol. For each visit date, BLIS computed the relative day of life (*DOL*) and the corrected gestational age (*DOL* + gestational age at birth). The protocol did provide several weeks of flexibility between participant *DOL* and *visit number*, so *DOL* was a foreign key for most tables, as well as a covariate for various analyses. *DOL* supported analyses on the effect of postnatal exposures, while the corrected gestational age was useful to examine effects of prematurity. Thus, every repeated-measure covariate and *biospecimen* (itself uniquely keyed) had the *participant ID*, *visit number*, *date*, *DOL*, and corrected gestational age associated with it.

The PROP and PRISM studies examined risk factors for growth failure and persistent respiratory disease, both chronic conditions that require longitudinal data to even diagnose them. To join data across domains in this longitudinal setting, such microbiome and clinical measurements, patient-reported outcomes, or immunological assays required fastidious attention to schema and identifiers. However, once this work was complete, we integrated microbiome measurements from multiple body sites, flow cytometry-base T cell assays, and clinical data often by simply joining on *visit number* and *participant ID*, sometimes in conjunction with simple data imputation techniques for data sampled on an irregular grid, such as last-observation-carried-forward. This analyses revealed evidence for microbial interaction between body sites, predictive in cross-validation, even after stringent control for the effect of host development [26], as well as a subset of T cells that were associated with inflammatory insults that occurred as early as birth [29].

The database schemas implied a number of invariant relationships between variables. We can catch many data errors and omissions by verifying these relationships using continuous quality control (no. 3). Ideally, automated methods connect laboratory or clinical data collection workflows to the database. This ensures that continuous quality control of the data occurs as data are generated, so that problems can be documented and resolved by the laboratory or clinical staff in a timely manner.

In BLIS, we automatically imported clinical data every night from REDCap [18] and specimen processing and inventory from a lab information management system. After integrating these data sources, their consistency was verified using the *participant ID*, *visit number*, *date* and clinical sample collection metadata. Data discrepancies, including data entry errors, would be reported out automatically.

For molecular and device results from the lab, we used a semi-automatic import and parsing of assay results, and instrument-generated data files were implemented in BLIS. Labs entered the *biospecimen* ID in assay software to ensure the resulting data files streamed from the instrument are uniquely identified. For instance, flow cytometry FCS file keywords and sequencing BAM file headers contained *biospecimen* ID, while for other results it was encoded in the file name. The *biospecimen* ID in the assay data was then crosschecked with the inventory of physical vials to verify that the vial did previously exist and had been consumed.

Shared team access to the cycles in Fig. 1a means that data and analyses should be access controlled, audited, and versioned (no. 4). Ideally, this allows those involved in a cycle to access and import data as necessary to repeat historical analysis, so that the implications of changes to the tables can be understood and errors bisected. Since the schema and fields can change as data are collected, quality evaluated, and clinical and laboratory workflows evolve, all data, original and derived, should be audited and versioned using appropriate change data capture methodologies.

In BLIS, we utilized the security and access controls and auditing capabilities built into the LabKey platform. In particular, datatable and access are designed with the principle of *least privilege*, meaning users have only enough access to do their job. Thus, lab technicians performing experiments do not have access to clinical data. Conversely, study coordinators do not have access to assay results while data collection is ongoing. Data engineers implement versioning procedures directly in the schema through versioning fields (applying *slowly changing dimensions* methodology Type 2), missing data codes, and frozen snapshots of individual tables or multiple subsets for specific analyses and historical reporting (*slowly changing dimensions* methodology Type 4). For the PROP and PRISM studies, flow cytometry experiments had up to four distinct versions, corresponding to various manual gating strategies or unsupervised clustering algorithms under evaluation. Our version control practices and the use of the wiki features increased transparency and clarified provenance to facilitate data reuse and team collaboration. Our servers were professionally managed and audited annually under our Information Technology Security Plan.

User-driven data exploration (no. 5) lowers the bar for interacting with the database, aiding in accelerating the research and knowledge exchange. Most members of a clinical or translational project are not computer programmers but still need to be able to query data. Data exploration can be used by the clinical and laboratory staff and analysts for quality control of clinical and assay data to generate counts of available data and samples for assay planning and logistics or for initial hypothesis generation.

In BLIS, we exploited LabKey’s spreadsheet-like operations of simple visualizations, distributional statistics, and boolean filtering which all can be exposed to the user. We also built custom reports and visualizations in either R and javascript and ran them as plugins in the system. Regardless of the data exploration method used, all visualizations can be saved and shared with other team members. These capabilities accelerated the research and knowledge exchange including [21]. These findings also spawned several successful grant applications, trainee funding, as well as pilot data used outside of the study labs for funding.

Derived variables should be imported (no. 6) and integrated into the schema. Modeling often depends on summarization, normalization, or other computation on collected data that generate new variables. Systematically importing these derived variables makes them centrally available, where they can be included in the “visualize–communicate–reuse” path. This promotes collaboration, increases efficiency, reproducibility, and traceability.

In BLIS, we exploited this in both simple and more complicated ways. We derived and imported temporal variables relative to a subject’s birth, by calculating the day of life and corrected gestational age when an event occurred. Besides saving users from implementing their own date calculus, which is notoriously difficult, exact event dates could be suppressed for most users, eliminating some risks of subject reidentification. More complicated variables, such as exposure indicators and respiratory outcomes, were calculated by algorithms we implemented. For high-throughput assays, important results from the computational pipelines that process these assays were included. These included sequencing quality reports, flow cytometry gating hierarchies, 16S operational taxonomic unit (OTU) count tables, and alpha and beta diversity scores.

Since team members are iterating between importation and transformation of study data, the transform–model–visualize–communicate loop should be able to access data in stable formats and programming interfaces (no. 7). Minimally, users and scripts should be able to manually query the database and have results returned to them in an open format. Ideally, updates to the database will automatically propagate to downstream analyses. BLIS makes provisions for this by exposing the LabKey Application Programming Interfaces and their associated libraries. Bindings exist in R, SAS, Java, and Python. In addition, using the BLIS web interface, version-stamped data can be exported in open formats such as comma and tab separated files.

Lastly, it is imperative in a Team Data Science project to provide online documentation (no. 8) that is current and easily located. At a granular level, BLIS provides a data dictionary for all data fields available in the schema, and descriptions of specific tables can be attached to each table and field to document how the data was collected or generated. In addition, the study portal contains analysis-specific pages that document analysis plans. They can include direct links to the versioned, frozen sources of data used in each analysis, as well as external dependencies like git repositories or references to methods. The BLIS study portal also contains links to institutional file servers for protocol versions, standard operating procedures, and background publications.

## Discussion

In this article, we propose an idealized workflow for Team Data Science. It modifies established workflows that consider data scientists in isolation, by adding connections between steps that respect the active and interdisciplinary nature of clinical and translational research. It addresses what we believe is the ultimate goal of this research: to improve human health by enabling reuse of data and models by the scientific community. Our workflow also recognizes that study members tend to interact centrally with some phases of the workflow, but often need to access other phases to do their jobs. Enabling good habits for even indirect use of the data science workflow is beneficial.

To maximize the speed and accuracy of applications of data science to clinical and translational projects, we describe eight principles and practices. Many of these are technical engineering decisions made when designing and implementing the database. Yet others, such as active collaboration, make demands on the overall management and provisioning of the study. However, even practices that seem essentially internal to database design are motivated by having it serve not just data scientists and engineers, but all study personnel. These practices include user-driven exploration, import of derived variables, and online documentation. We illustrate how we have applied these eight practices in BLIS, our management system for managing the data from several complex longitudinal studies run at the University of Rochester.

As both principal investigators and data scientists can attest, it is difficult to manage and coordinate the decentralized and interdisciplinary teams that large studies entail. It can be a challenge to answer even basic questions, such as “where are the data from my experiment?,” “what’s the latest version?” or “how can I link data between assays from the same subject?” Studies have often relied on “data shamans” to be the keeper of this knowledge. This is inefficient, since knowledge remains siloed, as well as fragile, since the knowledge can disappear with staff turnover. A remedy is to increase data stewardship maturity [30]. Put simply, this means replacing ad hoc approaches with systematic processes. The eight practices we describe represent some steps a study can take toward more systematic and mature data stewardship.

It should be acknowledged that the BLIS management system began as a dedicated Data Management and Biostatistics core as part of several NIAID-funded centers including the Center for Biodefense Immune Modeling, the New York Influenza Center of Excellence, and the Respiratory Pathogens Research Center, which provided a well-defined governance structure and crucially, initial funding for this informatics infrastructure. The BLIS management core leadership ultimately reported to the Research Center principal investigators but otherwise had flexibility in technical decision-making.

In this work, we focused on the data management for use by the initiating studies. We acknowledge that broader reuse of research data is critically important. To that end, we are continually depositing data in the NIH data repositories dbGaP and SRA under the accession numbers phs001297 and phs001347. As the complexity of data collected continues to evolve, the systems to capture and integrate these data must as well. We continue to seek efficiencies in the iterative import–tidy–transform phases by enabling integration of additional sources of clinical information and their associated data standards, vocabularies, and ontologies (e.g., ICD-10, RXNorm, LOINC). In our view, the greatest value in adopting common data models comes from the potential to harmonize multiple studies after completion [31]. Ideally, these standards will be introduced when the study is conceived and study coordinators and principal investigators can be coached to align their native vocabularies and representations with the common standard. However, it is also important to recognize that common models do not always faithfully represent all facts and relationships present in a particular study. In that case, multiple representations would need to be maintained, increasing costs and complexity. We suggest focusing on the data models and representations that have will have the largest scientific return on investment to a given study.

Data science has encouraged rapid prototyping of scientific hypotheses using advanced methods from statistics and machine learning. These have yielded impressive benefits to many areas. However, it is important to recognize and mitigate against the downsides of these techniques, which can be especially acute in observational studies. Rapid prototyping and reusability can lead to more overfitting of models and fishing for statistical significance. More generically, selection biases and unmeasured confounding will be present in all observational studies. Even if causal interpretations are not explicitly sought, it is still important to consider how selection bias and confounding impacts conclusions. Overall, we believe the solution to these pitfalls is not less usability, but more use of unbiased validation, and especially, more active collaboration between domain experts, data engineers, technicians, statisticians, and data scientists.
